# Effect of Ethanol-Derived Clove Leaf Extract on the Oxidative Stress Response in Yeast *Schizosaccharomyces pombe*

**DOI:** 10.1155/2019/2145378

**Published:** 2019-08-14

**Authors:** Anninda Faiz Fauzya, Rika Indri Astuti, Nisa Rachmania Mubarik

**Affiliations:** Department of Biology, Faculty of Mathematics and Natural Sciences, IPB University, IPB Dramaga Campus, Bogor, West Java 16680, Indonesia

## Abstract

Compared to the widely explored antioxidant activity from the clove bud extract, less data are available regarding the potential pharmacological use of clove leaves. Our study aimed to explore the antioxidant activity of clove leaves extract in the cellular level. Thus, we used the yeast *Schizosaccharomyces pombe* as model organisms. Our data indicate that, following extract treatment (100 ppm), the viability of the stationary phase cells of *S. pombe* was higher than without extract and that of calorie restriction treatments. 100 ppm extract treatment also increased cell viability against H_2_O_2_-induced oxidative stress. Those data indicate that the extract could promote oxidative stress tolerance response in yeast cells, which occurred either during the stationary phase or due to exogenous exposure. Higher dose of extract (500 ppm) showed opposite effects, as cell viability was lower than that without treatment. Analysis toward the mitochondrial activity revealed that the extract did not induce mitochondrial activity unlike the calorie restriction treatment. Based on our data, clove leaf extract promotes oxidative stress tolerance response in the yeast *S. pombe*, independent to that mitochondrial adaptive ROS signaling which commonly occurs in calorie restriction-induced oxidative stress tolerance response.

## 1. Introduction

Reactive oxygen species has been acknowledged as the main cause of oxidative stress [1]. Although the cells are capable of combating the accumulation of ROS via oxidative stress response, it is often insufficient and thus leads to the severe oxidative stress [2]. Such severe oxidative stresses conditions have been attributed to the prevalence of various cellular disorders and pathogenesis of diseases. For instance, oxidative stress is attributed to the genetic mutations [3], accumulation of toxic amyloid body [4], organelle dysfunction [5], and cellular aging [6]. Such events may culminate to the development of diseases including degenerative diseases and metabolic diseases such as diabetes, Parkinson, cancer, and Alzheimer diseases [4, 7, 8].

Antioxidant plays an important role in combating oxidative stress. Antioxidant properties from plant-derived flavonoid compounds have been widely studied as one of the strategies to induce and strengthen oxidative stress response in cellular systems. Amongst native Indonesian plants, clove (*Syzygium aromaticum*) is gaining serious attention due to its potential pharmaceutical uses. This plant represents one of the richest sources of phenolic compounds such as eugenol, tannins, and gallic acid that are potential for pharmaceutical and cosmetic uses [9]. Bioactive compounds from clove buds have been widely studied regarding its antioxidant activities. For instance, ethanol and aqueous extracts of clove buds could inhibit 95% activity of both radicals of superoxide and 2,2-diphenyl-1-picrylhydrazyl (DPPH) [10]. Further in vivo study revealed that eugenol plays a significant role in protecting male rats exposed to aflatoxins-induced hepato, nephrotoxicity, and oxidative stress [11]. In addition, extract from clove buds is applicable as food antioxidants, as applied for soybean oil [12] and sausages [13].

Study on the biological activity of clove extract from leaves is less available than that on the buds. Clove leaf has been used as source for clove essential oil. Jirovetz et al. [14] reported that clove leaf essential oil comprises more than 20 chemical compounds with eugenol (76.8%), followed by *β*-caryophyllene (17.4%), *α*-humulene (2.1%), and eugenyl acetate (1.2%) as the main components. On the other hand, clove bud essential oil mainly comprises eugenol (72%) but higher concentration of eugenyl acetate (8%) yet lesser *β*-caryophyllene (2.8%) than the bud-derived essential oil [15]. Clove leaf extract has been reported to have a radical scavenging activity based on the DPPH assay [14]. To date, information on the antioxidant activity of clove leaf extract in a cellular level has not been reported yet. A recent study by Munisa et al. [16] showed that methanol extract of clove leaf could stabilize enzymes, essential for oxidative stress response, i.e., Cu-SOD and Zn-SOD, as well as reduce malondialdehyde in the liver in a hypercholesterolemia rabbit. To further understand the mode of action of clove leaf extract in vivo, we used the fission yeast *Schizosaccharomyces pombe* as model organisms. In this study, we aimed to analyze the effect of ethanol extract of clove leaf on a cellular system, especially toward cell response against oxidative stress.

## 2. Method

### 2.1. Clove, Strains, and Medium

The clove leaves (*Syzygium aromaticum* var Zanzibar) were obtained from the Indonesian Spice and Medicinal Crops Research Institute (ISMCRI). The fission yeast *S. pombe* ARC 039 (*h*^*−*^*leu1-32 ura4-294*) was used as the model organism. *S. pombe* was routinely maintained in Yeast Extract with Supplement (YES) medium. YES (1 L) composition is as follows: 5 g yeast extract, 20 g glucose, 0.128 g histidine, 0.128 leucine, 0.128 adenine, 0.010 g uracil, and 0.128 arginine.

### 2.2. Extraction of Clove

The extraction was conducted based on the previous method as described by Cortés-Rojas et al. [9]. The clove leaves were macerated using 70% ethanol at a ratio of 1 : 5 (sample : solvent). The sample was soaked for 24 hours and stirred at each 12 hours. Following the harvest of the macerated substance, the process was repeated twice by using the same volume of solvent. Substance was then collected and concentrated by using the rotary evaporator (45°C) within one hour. The ethanol-derived extract was obtained in paste with dark brown color.

### 2.3. Quantitation of Flavonoid

Flavonoid content was measured via the aluminium chloride colorimetric assay. Quercetin was used as the reference compound. For prior measurement, ethyl acetate fraction of crude extract was prepared by using series of solvents. In brief, crude extract (∼200 mg simplisia) was suspended in 1 ml of 0.5% hexamethylenetetramine, 20 ml acetone, and 2 ml HCL. Solutions were then hydrolyzed for 30 minutes and then filtered. The residue was then distilled with addition of 20 ml acetone for 30 minutes. The filtrate was then suspended in 100 ml acetone. About 20 ml suspension was then extracted three times with 15 ml ethyl acetate solutions to obtain the ethyl acetate fractions. 1 ml of 10% AlCl_3_ was added to the ethyl acetate fractions. Acetic acid glacial solutions were subsequently added to adjust final volume of 25 ml. The mixture was allowed to stand for 30 minutes, and absorbance was measured at 510 nm. The total flavonoids content was expressed as mg E quercetin/g extract.

### 2.4. Cell Viability Assay of Stationary Phase Cells

The cell viability test was conducted via the spot assay. Culture of yeast was prepared by inoculating one loop of yeast colony in YES liquid medium. Culture was then incubated for 24 hours in a shaker at room temperature and used as subculture. Turbidity of the subculture was then measured at 600 nm by using the spectrophotometer. The subculture was then transferred to another YES liquid medium in a final OD_600_ = 0.1 to prepare main cultures. Clove leaf extract was added into the main culture at various concentrations including 100 ppm, 200 ppm, and 500 ppm. Negative control treatment was performed by adding extraction solvent (DMSO) but without extract, instead. The positive control was prepared by culturing yeast cells in YES medium with lower glucose concentration (0.5% glucose) to develop calorie-restriction (CR) conditions. CR was used as positive control treatment since such conditions has been reported to increase the yeast chronological life span by inducing oxidative stress responses [17], autophagy [18, 19], and mitochondrial biogenesis [20]. It is also believed that mammalian cells regulate the same mechanisms following CR conditions to extend longevity of the cells and prevent degenerative diseases [21, 22].

Main cultures were incubated for nine days in a shaker at room temperature. Every three days, the culture was harvested for spot assay. OD_600_ of each culture was measured by using the spectrophotometer. Specific volume of each main culture was transferred and then adjusted to final OD_600_ = 1 in sterile water to a final volume of 200 *μ*l. Cell suspension was then serially diluted to 10^4^ dilutions. About 3 *μ*l of each dilution was then spotted on top of YES agar and further incubated at 30°C for three days.

### 2.5. Cell Viability Assay against Oxidative Stress

For this assay, main cultures were prepared as described above in the cell viability assay of stationary phase cells. YES agar containing H_2_O_2_ (1, 2 and 3 mM) was used for spot assay, instead.

### 2.6. Mitochondrial Analysis

The procedure was done following [17]. Main cultures were prepared as described in the previous assays. Following the overnight incubation of main cultures, the pellet cells were harvested by using 0.05 M phosphate buffer (pH 7.4). The cell pellet was then suspended in 1 ml of the same buffer and added with 10 *μ*l of 100 nM rhodamine B. The cell mixture was allowed to stand for 30 minutes at 25°C. Light exposure was avoided throughout the experiment. The mitochondrial activity was observed under a fluorescence microscope (Olympus BX51).

## 3. Results and Discussion

### 3.1. Flavonoid Content

The yield of ethanol clove extract from 500 g of clove leaf was 64.6 g (12.9%). Based on the quercetin standard curve, the flavonoid content of clove leaf was 99.6 mg·QE/100 g extract. Compared to other spices, the ethanol-derived clove leaf extract contained high flavonoid concentrations. For instance, by using 80% ethanol, the flavonoid content of torch ginger and lemon grass was 39.7 mg·QE/100 g extract and 14.8 mg·QE/100 g extract, respectively, much lower than the clove leaf extract. However, the flavonoid content of clove leaf extract was lower than that of curry leaf (144.5 mg·QE/100 g) by using the same solvent [23]. Our data indicated that the flavonoid content of clove leaf and bud was not significantly different (unpublished data).

### 3.2. Cell Viability Assay of Stationary Phase Cells

**Figure 1:**
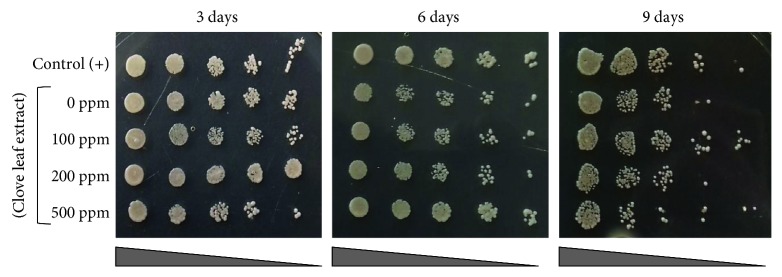
Effect of clove leaf extract on the cell viability of the yeast *S. pombe* during the stationary phase. Cell viability was assayed by using the spot assay. Yeast cells were cultured for nine days in YES liquid medium supplemented with 100, 200, and 500 ppm of clove leaf extract. Every 3, 6, and 9 days of incubation, yeast cells were spotted on top of YES agar medium and incubated for three days at 30°C. The culture of yeast in YES medium without supplementation of clove leaf extract (0 ppm) was used as negative control. Yeast cells grown in YES medium with lower glucose concentration (0.5% glucose) was used as positive control.

In this assay, we attempted to evaluate the effect of clove leaf extract in the cell viability of yeast cells during stationary phase cells. Treatment of 100 mM clove leaf extract promotes cell viability of yeast, which is clearly seen in the aging culture (9 days of incubation), compared to that without extract and other clove leaf extract treatments (Figure 1). Interestingly, the particular treatment resulted in higher cell viability compared to calorie restriction treatments (Figure 1).

Stationary phase cells are frequently used to assay the chronological life span of yeast as they mimic the most spent conditions of postmitotic tissue of higher organisms [24]. In the stationary phase, yeast mostly uses mitochondrial respiration to gain energy [25], which consequently leads to the accumulation of intracellular ROS. Therefore, stationary phase yeast cells are more tolerant against oxidative stress compared to the log phase cells, since the former phase could develop adaptive mitochondrial ROS signaling [26]. Such adaptive mitochondrial ROS signaling was also involved in the oxidative stress tolerance mechanism of CR-cells [27]. Addition of clove leaf extract surprisingly increased the cell viability in the aged yeast cells, even higher to those CR-cells. Such data indicate that clove leaf extract is likely capable of modulating oxidative stress response in a way that is more effective than CR conditions. A previous study had reported that in addition to the autophagy and mitochondrial activities, the dynamic actin cytoskeleton is involved in the survival of stationary phase, thus extending the life span of the yeast *S. cerevisiae* [28]. To our knowledge, this is the first report of the use of clove leaf extract to promote cell viability of yeast cells, which suggest its potential as an antiaging agent.

### 3.3. Cell Viability Assay against H_2_O_2_-Induced Oxidative Stress

**Figure 2:**
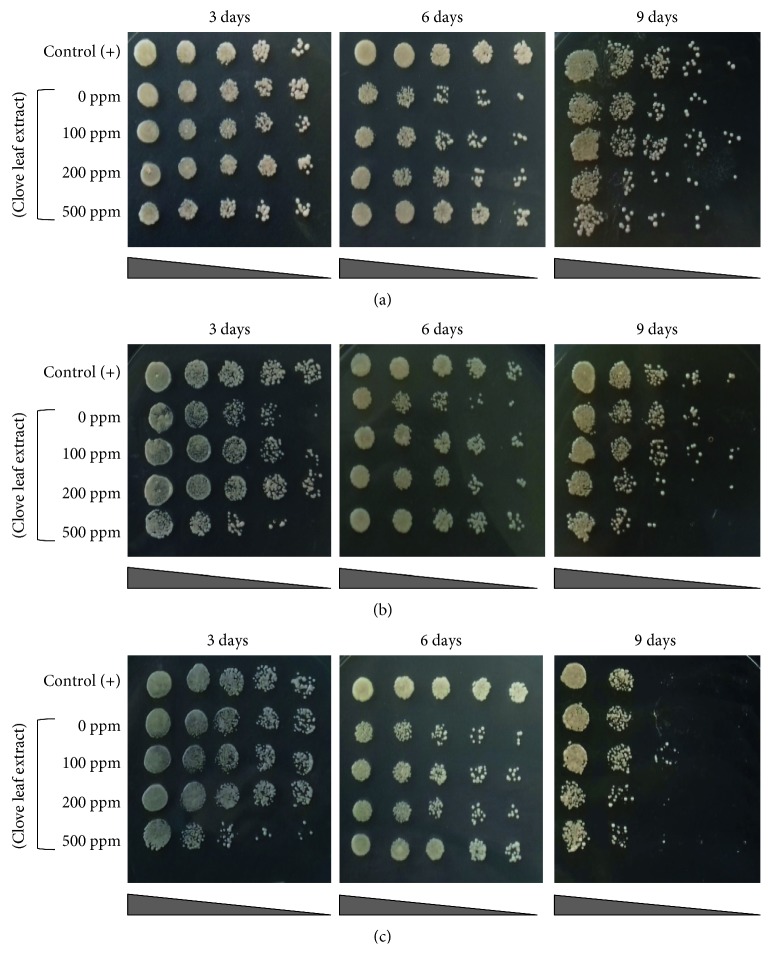
Effect of clove leaf extract on the cell viability of the yeast *S. pombe* against H_2_O_2_-induced oxidative stress: (a) 1 mM H_2_O_2_, (b) 2 mM H_2_O_2_, and (c) 3 mM H_2_O_2_. The cell viability was assayed by using the spot assay. Yeast cells were cultured for nine days in YES liquid medium supplemented with 100, 200, and 500 ppm of clove leaf extract. Every 3, 6, and 9 days of incubation, yeast cells were spotted on the top of YES agar medium containing H_2_O_2_ and incubated for three days at 30°C. The culture of yeast in YES medium without supplementation of clove leaf extract (0 ppm) was used as negative control. Yeast cells grown in YES medium with lower glucose concentration (0.5% glucose) was used as positive control.

To further understand the effect of clove leaf extract in the oxidative stress response, we assayed cell viability against H_2_O_2_-induced oxidative stress conditions. In line with the previous results, 100 ppm of clove leaf extract treatment enhanced cell viability of aging culture (9 days) against moderate (Figure 2(a)) and severe oxidative stress exposures (Figures 2(b) and 2(c)). It is worth noting that treatment of higher concentration of clove leaf extract (200 and 500 ppm) showed opposite phenotype to the 100 ppm and control treatments (Figures 2(b) and 2(c)). These data indicate that the effect of clove leaf extract in promoting oxidative stress response in yeast is unlikely to occur in a dose dependent manner.

Following moderate oxidative stress exposure (1 mM H_2_O_2_), the early stationary phase cells (3 days of age) were unaffected by oxidative stress exposure (Figure 2(a)). However, in severe oxidative stress treatment (2 and 3 mM H_2_O_2_), three days of age cells already showed growth defect, especially the negative control and high extract treatments (Figures 2(b) and 2(c)). In aged cells (9 days of age), oxidative stress caused defect in cell viability (Figures 2(a)–2(c)), yet only 100 ppm extract treatment resulted oxidative stress tolerance phenotype (Figures 2(a)–2(c)).

Our data suggest that clove leaf extract could promote oxidative stress tolerance in yeast cells. Other extracts that are capable of inducing oxidative stress tolerance response in yeast are green tea extract [29], Red chicory leaf [30], *Tinospora cordifolia* [31], and roselle petal [32].

Mechanisms of oxidative response in yeast are complex. Various transcriptional factors have been reported to regulate oxidative response. For instance, in the yeast *S. pombe*, transcriptional factors Pap1 and Sty1 are responsible for upregulating genes involving in oxidative stress responses against low and high H_2_O_2_ levels, respectively [33]. Among genes under the regulation of Pap1 and Sty1 are *ctt1, sod1, srx1, trx1*, etc [34, 35]. The molecular mechanism of green tea extract to modulate oxidative stress response in *Saccharomyces cerevisiae* is occurred via upregulation of transcriptional Yap1 [29], Pap1 homolog in *S. pombe*. On the other hand, direct ROS scavenging activity was shown by phyotextract from *Tinospora cordifolia* [31]. In a calorie restriction-induced oxidative response, activation of the mitochondria promotes adaptive ROS signaling, thus increasing the stress tolerance. We further analyze the effect of clove leaf extract on the mitochondrial activity.

### 3.4. Mitochondrial Analysis

**Figure 3:**
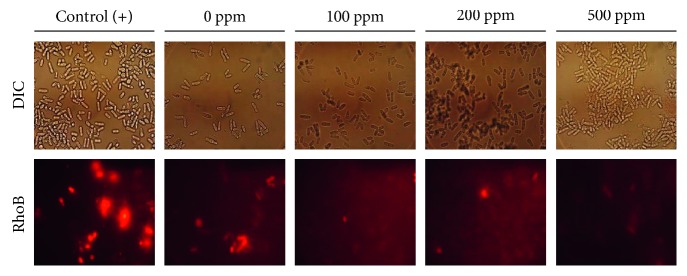
Effect of clove leaf extract on mitochondrial activity of the yeast *S. pombe*. Mitochondrial activity was assayed by using probe rhodamine B. Yeast cells were cultured for nine days in YES liquid medium supplemented with 100, 200, and 500 ppm of clove leaf extract. The yeast culture in YES medium without supplementation of clove leaf extract (0 ppm) was used as negative control. Yeast cells grown in YES medium with lower glucose concentration (0.5% glucose) was used as positive control.

The mitochondrial activity was assayed by using rhodamine B. Strong or bright red color intensity represents active mitochondria. Treatment of clove leaf extract did not increase mitochondrial activity (Figure 3). Calorie restriction treatment significantly induced the mitochondrial activity, as reported elsewhere [20, 36]. Interestingly, the mitochondrial activity was unaffected by the clove leaf extract. Our data suggest that clove leaf extract-induced oxidative stress tolerance is unlikely to occur via mitochondrial adaptive ROS signaling, as occurred in calorie restriction conditions.

The antioxidant activity of clove leaf extract might have happened through the direct ROS scavenging activity. To understand better the mode of action of clove leaf extract, we attempted to measure the intracellular ROS level in the yeast *S. cerevisiae* following the oxidative stress exposure.

### 3.5. Intracellular ROS Levels

**Figure 4:**
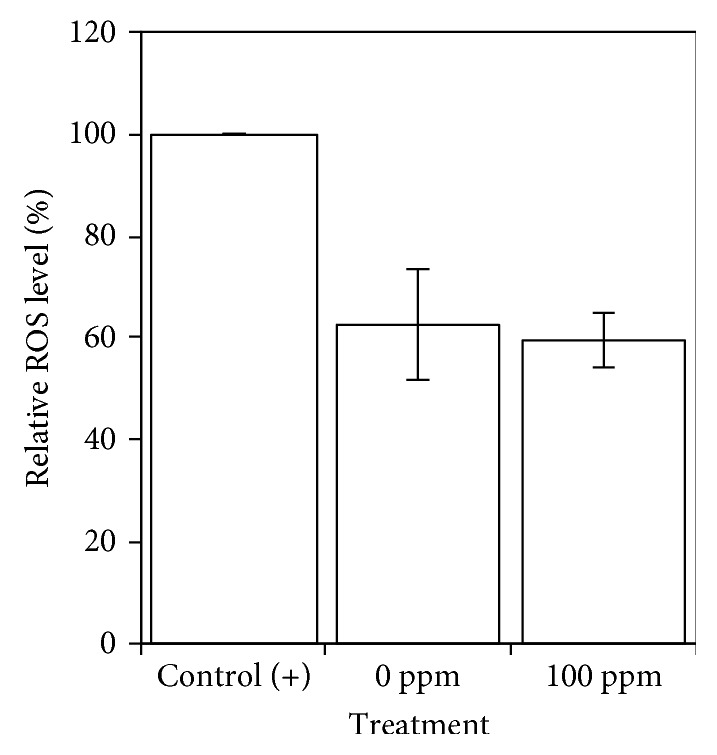
Effect of clove leaf extract on the yeast intracellular ROS level. Mitochondrial activity was assayed by using the probe. Rhodamine B yeast cells were cultured for nine days in YES liquid medium supplemented with 100, 200, and 500 ppm of clove leaf extract. The culture of yeast in YES medium without supplementation of clove leaf extract (0 ppm) was used as negative control. Yeast cells grown in YES medium with lower glucose concentration (0.5% glucose) was used as positive control.

Treatment of clove leaf extract lowered intracellular ROS levels in yeast under oxidative stress conditions, as compared to those CR conditions. In negative control treatment, however, the intracellular ROS level was not significantly different from those extract-treated cells (Figure 4). These data support the fact that 100 ppm of clove leaf extract is not in a pro-oxidant mode of actions. Furthermore, it is likely that addition of clove leaf extract effectively induces ROS scavenging activity, either by direct or indirect mechanisms.

Plant-derived natural antioxidant molecules, such as flavonoids, have been reported to act as free-radical scavengers, reducing agents, complexes of pro-oxidant metals, and quenchers of singlet oxygen and thus often used as supplementary dietary intake to deal with oxidative stress-induced diseases [37]. For instance, flavonoid substances in the methanolic extract of Indian medicinal plants, *Caesalpinia crista* Linn. (syn. *C. bonducella* [L.]. Roxb.) (family Fabaceae) exert direct effect via ROS scavenging and iron-chelating activities in vitro and in vivo [38]. On the other hand, a study on the ethanol extract of *Pterygota alata* shows its potential indirect antioxidant activity via induction of the intracellular antioxidative stress enzyme, superoxide dismutase, in a liver-damaged mice [39]. Further research in revealing the mode of actions from flavonoid substances of clove leaf extract is needed to clarify the effect of the particular extract on cellular and molecular levels.

## 4. Conclusions

Clove leaf extract treatment could promote the oxidative stress tolerance phenotype in the yeast *S. pombe* that exerts not in a dose-dependent manner. Optimum clove leaf extract (100 ppm) shows greater effect on the cell viability of stationary phase cells in oxidative stress conditions than that in calorie-restriction conditions. The mitochondrial activity is unaffected by clove leaf extract, suggesting that the yeast tolerance against oxidative stress is mitochondrial adaptive ROS signaling independent, but via direct ROS scavenging activity, instead.

## Data Availability

The data used to support this study are provided within the article.
